# Racism and Structural Violence: Interconnected Threats to Health Equity

**DOI:** 10.3389/fpubh.2021.676783

**Published:** 2022-02-03

**Authors:** Mienah Zulfacar Sharif, Jennifer J. García, Uchechi Mitchell, Elinam D. Dellor, Natalie J. Bradford, Mandy Truong

**Affiliations:** ^1^Department of Epidemiology, University of Washington, Seattle, WA, United States; ^2^Center for the Study of Racism, Social Justice and Health, University of California, Los Angeles (UCLA), Los Angeles, CA, United States; ^3^Engage R+D, Pasadena, CA, United States; ^4^Department of Community Health Sciences, University of Illinois at Chicago, Chicago, IL, United States; ^5^College of Social Work, The Ohio State University, Columbus, OH, United States; ^6^Department of Health Policy & Management, University of California, Los Angeles Fielding School of Public Health, Los Angeles, CA, United States; ^7^School of Nursing and Midwifery, Monash University, Clayton, VIC, Australia

**Keywords:** racism, equity, public health critical race praxis, violence, anti-Black racism, systems-level, social epidemiology

## Abstract

In 2020, the continuing murder of Black Americans by police officers received widespread media attention and sparked global outrage. Public health responses to these events focused on discrimination by police and structural racism in broader society. However, police violence is but one of many forms of racialized violence propagated by structural racism and anti-Black racism in particular. We aim to expand the current public health dialogue by describing how structural racism and structural violence are deeply interrelated; embedded in institutions, systems, and processes; and threaten health, safety, and well-being across the life course for racialized minority groups. Structural racism and structural violence are threats to health equity and anti-racist public health work.

“Until American society can commit to full racial justice and the abolishment of structural violence, people will continue to protest, disrupt, and demand. Toward this end, the charges we give to ourselves and the health professional community below are necessary first steps to stop and reverse the injustices that sit at the intersection of racism and violence.” – COVID-19 Task Force on Racism & Equity (1).

## Introduction

Within policy and scientific forums, there are ongoing conversations on whether America is a racist society and to what extent the country grapples with the presence and implications of racism. As a collective of women of color, anti-racist health scholars we unequivocally assert that America is, and has always been, a racist country. In fact, racism is quintessentially American. And America is an exceptionally violent society. These two truths are not often discussed in tandem, especially within dominant public health narratives. An equity-driven outlook recognizes that intersecting forms of oppression (e.g., racism, sexism, heterosexism, classism) have prevailed since the founding of the United States (U.S.). These structural inequities have become so deeply engrained within the country's social, political, and economic fabric and a timeline enumerating the many examples is beyond the scope of this paper. However, events within the last 12 months including the violent January 6^th^ attack on the U.S. Capitol, the disproportionate death rates due to COVID-19 in Indigenous, Black, and Pacific Islander communities (2), the excessive rates of fatal shootings by police officers in Black, Indigenous, and Latinx communities (3), and the rise in anti-Asian violence including physical assaults, harassment, and other hate incidents (4), are examples of violence fueled by white supremacy. Together, these recent events and a historical perspective, lay bare that racism and violence are deeply intertwined and normalized facets of everyday life that prevail in both overt and covert forms to harm and threaten the lives of racially marginalized populations. In this article, we underscore the need for the field of public health to adopt a broader understanding of structural racism and structural violence in order to adequately address health inequity.

Police violence is increasingly recognized as a public health issue (5). However, police violence is but one of many forms of racialized violence propagated by structural racism and anti-Black racism in particular. The symbiotic relationship between structural racism and structural violence is long standing with origins dating back to European colonialism. Throughout history and across global contexts, structural violence is the mechanism that carries white supremacist principles forward, impeding health equity through genocide, human rights violations and widespread detainment, and exploitation of people of color. Together, structural racism and violence have created, sustained, and exacerbated social, economic, and health inequities and influence policies, practices, and scientific knowledge production processes (6, 7). To confront these fundamental barriers to health equity, public health's conceptualization and analysis of structural forms of racism and violence must be broadened. Furthermore, an intersectional perspective is required to elucidate the ways racism and violence intersect with various social locations (e.g., gender, sexual orientation, and class) to create different health risks and consequences, particularly among marginalized groups (8). While this paper focuses on the U.S. context, the racist and violent underpinnings of American policies (both domestic and foreign) have global implications that undermine safety and health among some of the most vulnerable populations and continue to uphold oppressive systems and structures around the world.

## Defining Structural Racism and Structural Violence

The field of public health is charged with the ultimate goal of pursuing health equity, defined as the “assurance of the conditions for optimal health for all people” (9). To address the roots of health inequities, we encourage the field to adopt explicitly structural definitions of racism and violence. For example, Ruth Wilson Gilmore describes racism as “the state-sanctioned or extralegal production and exploitation of group-differentiated vulnerability to premature death” (10). This definition is particularly helpful for viewing racism through a structural lens (i.e., as a product of state-sanctioned and extralegal exploitation) and underscores the central role of structural racism in perpetuating health inequities (i.e., group-differentiated vulnerability to premature death). Gilmore's definition clearly ties racism to health inequities and life-threatening harm. As H. Jack Geiger explained nearly 25 years ago, it is this “result in predictable harm to the physical and mental health of large populations” that makes structural racism inherently violent (11).

In a recent commentary on the history of medical racism and violence in the U.S. (12), Paul Farmer et al. define structural violence as “social arrangements that put individuals and populations in harm's way. The arrangements are *structural* because they are embedded in the political and economic organization of our social world; they are *violent* because they cause injury to people” (13). Despite efforts to examine violence as a structural concept (14), the public health field tends to understand violence as a problem of individual crime, behavior, and risk, which is evident in conventional violence prevention frameworks. However, like racism (15), violence operates on multiple levels. A structural analysis acknowledges that racism and violence do not solely or primarily depend on the intentions or (in)actions of individuals. Rather, the damaging manifestations of structural racism and violence are often the consequence of white supremacist ideologies entrenched in (and across) systems (e.g., education, housing, healthcare, immigration systems).

Racial inequities in health are exacerbated by capitalism, an economic system that has historically exploited people of color. Capital (e.g., land, resources, labor) continues to be accumulated through violent means—war, colonization, seizure and dispossession of land—and is central to systems of slavery, segregation, genocide, incarceration and migrant exploitation (11, 16, 17). Racial capitalism disproportionately exposes communities of color to environmental hazards, insufficient funding for resources such as schools and parks, limited healthy food options, and inadequate access to healthcare services (18). In short, racial capitalism itself is a form of structural violence that bolsters the social and commercial determinants of health inequities. Racism and violence are so deeply embedded within structures that they are difficult to recognize because they have become normalized. In other words, structural racism and violence are foundational to the creation, maintenance, and normal operation of systems within which people live.

Health equity scholars, practitioners and policymakers must constantly ask and answer the question, “How are racism and violence operating here?”, “In what ways do our research, practices, policies, and norms disproportionately harm people and communities of color and disproportionately benefit people and communities racially classified as white?” As Camara Jones suggests, addressing questions like these “can be a powerful approach to identifying levers for potential intervention” (19). In the next section we describe four examples of the intersection between racism, violence, and health: 1) the education system, 2) child welfare systems, 3) the weaponization of data, and 4) predictive technologies.

## Structural Racism and Structural Violence Operating Across Interconnected Systems

### Education

State-sanctioned violence against Black communities extends beyond the carceral state (e.g., criminalization, mass incarceration and hyper-surveillance) (20, 21) and operates through systems of education (22) and child welfare (23). For instance, the presence of law enforcement in schools has permitted police to violently discipline children. This practice not only physically and mentally hurts children, it has resulted in the normalization of the school-to-prison pipeline and the disproportionate arrest of Black students (24). Student arrests are associated with an increased risk of continued contact with law enforcement and the judicial system (25), lower likelihood of graduating from high school (26) and lower employment opportunities (27); all of which have short- and long-term social and economic ramifications that undermine the health of Black youth and families (28).

### Child Welfare

For its role in disrupting Black families and inflicting racial harm on poor people of color (29), the child welfare system and mandated reporting procedures constitute “policing by another name” (30). Black children and families have been overrepresented in the child welfare system for over 50 years and experience worse outcomes than their white counterparts in every major case decision and milestone. For example, Black children are more likely to be separated from their families and placed in foster care and less likely to be reunified with their biological parents (31). Although explanations for the disproportionate representation of Black children in the child welfare system are multifactorial, Dettlaff and Boyd call out “structural and institutional racism, both within the child welfare systems and society at large” (pp. 257) as the fundamental cause (32). The cumulative effects of the physical and psychological trauma Black youth face at the hands of these systems may continue well into adulthood and old age.

### Data Weaponization

The weaponization of health data refers to the ways data collection, analysis, interpretation, and application are used to harm and exploit people. Numerous COVID-19-related examples of data weaponization against communities of color exist. For one, public health agencies rarely collect or share comprehensive race data on COVID-19 cases, testing, hospitalizations, and fatalities (33). As Indigenous Peoples and Indigenous Studies scholars have repeatedly and thoroughly explained, the lack of data on racialized groups “effectively erases their existence” (34). This data invisibility is a form of erasure, and “erasure is a kind of violence, and violence is one of the tenets of white supremacy” (35). A second example of data weaponization in public health is the traditional epidemiologic frame that focuses on an individual's biological and behavioral risk factors rather than the structural conditions that drive differential health outcomes. Without a critical and race-conscious perspective (36), narratives about COVID-19 that emphasize “pre-existing medical conditions” among Black, Latinx, and Indigenous communities obscures the role that systems, institutions, and practices play in increasing their odds of contracting the virus (37, 38).

### Predictive Technologies

Predictive technologies represent a more contemporary form of structural violence. Increasingly health scientists are developing and using advanced technologies including artificial intelligence (AI), machine learning, and algorithmic tools. At face value, these technological advancements have the potential to enhance our prediction of social phenomena. However, as with any statistical model, they can also reproduce bias (39). Colorblind coding schemas employ proxy indicators (e.g., educational status) that are emblematic of racialized social hierarchies, a practice sociologist Ruha Benjamin coined the “New Jim Code” (40). This is concerning given the wide use of algorithms across public and private sectors including immigration agencies, healthcare, and criminal justice. The current COVID-19 context presents another timely and concerning example of the uncritical adoption of new technologies. Blanket statements, even among health disparities researchers, for “more data” and contact tracing lack an understanding of: 1) how communities of color are threatened by ongoing, excessive forms of hyper-surveillance and 2) how the lack of an equity-driven approach to data collection and data sharing across governmental agencies can threaten the health and safety of racialized communities. Like other forms of structural racism and violence, the presence and utilization of these technologies have become deeply embedded and normalized in our processes and institutions to the extent that many of us are unaware of their presence in our daily lives nor how they further perpetuate racial inequities in health.

## Conclusion

Structural racism and violence take on many forms, evolving over time and becoming harder to detect; they easily become institutionalized and consequently invisible to the uncritical eye and thus unchallenged. The invisibility and ordinariness of these structures bolsters white supremacy and further harms marginalized communities (41).

The work of addressing structural racism and structural violence is a constant and collective struggle. To directly engage in and support anti-racist and anti-violence initiatives it is imperative that we develop equitable and interdisciplinary partnerships beyond the academy; collaborations that are not “solely for the purpose of data collection” (42), but to understand and intervene on the “levers” at play that Jones describes. The public health community must learn from collectives such as Black Lives Matter (43), Cops Off Campus (44), and Critical Resistance (45) about the very real harm that current systems, policies, and practices (e.g., criminalization of school discipline) inflict on communities of color and importantly, about alternative strategies to counter violence and build healthy and safe communities through abolitionist movements. Similarly, public health researchers and practitioners can follow the lead of data scientists, racial justice activists, and community organizers who use data as a tool for liberation and social change. The Algorithmic Justice League (46), Data for Black Lives (47), Stop AAPI Hate (48), Stop LAPD Spying Coalition (49), and We All Count (50), that advocate for data transparency and accountability, and engage in the critical and intentional use of data for social justice action (e.g., gather disaggregated race data, disseminate findings to impacted communities, etc.). These are just a few examples of where we can thoughtfully and intentionally engage in partnership and solidarity to dismantle structural racism and structural violence and move us closer toward the goal of equity.

A broader conceptualization of violence and a comprehensive understanding of the interrelationship between racism and violence is long overdue. Until the public health community acknowledges the centrality of structural racism and its inherent violence, we will consistently fall short of our health equity goals and social justice mandate.

## Data Availability Statement

The original contributions presented in the study are included in the article/supplementary material, further inquiries can be directed to the corresponding author/s.

## Author Contributions

All authors listed have made a substantial, direct, and intellectual contribution to the work and approved it for publication.

## Conflict of Interest

The authors declare that the research was conducted in the absence of any commercial or financial relationships that could be construed as a potential conflict of interest.

## Publisher's Note

All claims expressed in this article are solely those of the authors and do not necessarily represent those of their affiliated organizations, or those of the publisher, the editors and the reviewers. Any product that may be evaluated in this article, or claim that may be made by its manufacturer, is not guaranteed or endorsed by the publisher.
